# Can a data driven obesity classification system identify those at risk of severe COVID-19 in the UK Biobank cohort study?

**DOI:** 10.1038/s41366-021-00891-6

**Published:** 2021-07-06

**Authors:** Stephen Clark, Michelle Morris, Nik Lomax, Mark Birkin

**Affiliations:** 1 Consumer Data Research Centre and School of Geography, University of Leeds, LEEDS, LS2 9JT UK; 2 School of Medicine and Consumer Data Research Centre, University of Leeds, LEEDS, LS2 9JT UK; 3 School of Geography and Consumer Data Research Centre, University of Leeds, LEEDS, LS2 9JT UK; 4 Consumer Data Research Centre and School of Geography, University of Leeds, LEEDS, LS2 9JT UK

**Keywords:** Risk factors, Health policy

## Abstract

COVID-19 is a disease that has been shown to have outcomes that vary by certain socio-demographic and socio-economic groups. It is increasingly important that an understanding of these outcomes should be derived not from the consideration of one aspect, but by a more multi-faceted understanding of the individual. In this study use is made of a recent obesity driven classification of participants in the United Kingdom Biobank (UKB) to identify trends in COVID-19 outcomes. This classification is informed by a recently created obesity systems map, and the COVID-19 outcomes are: undertaking a test, a positive test, hospitalisation and mortality. It is demonstrated that the classification is able to identify meaningful differentials in these outcomes. This more holistic approach is recommended for identification and prioritisation of COVID-19 risk and possible long-COVID determination.

## Introduction

The COVID-19 pandemic has impacted lives globally. As healthcare professionals and scientists learn more about the disease, it becomes clearer that certain groups of people suffer more serious consequences of COVID-19. People who are overweight or living with obesity fare particularly badly. Above average prevalence of overweight and obesity is observed in patients requiring intensive care treatment [1–3]. Furthermore, the severe consequences of COVID-19 are impacting most upon adults over the age of 50, those from ethnic minority groups and the less affluent in society [3].

Drivers of obesity are complex and multifaceted [4], making the prevention and treatment for obesity challenging for all involved, from global organisations, through to the individual. It also makes relationships between COVID-19 and obesity extremely difficult to unpack. For example: are the severe consequences due solely to the weight status of an individual? Or is it weight status in combination with related comorbidities [5, 6]? Alternatively, are all of these driven by deprivation, [7], where higher incidence of severe COVID-19 symptoms are observed [3].

The United Kingdom Biobank (UKB) [8] has made COVID-19 relevant data available to researchers actively working with UKB data [9]. These include information on test results, primary care data, hospital admission data and mortality for UKB participants. The timeliness of these data has allowed a body of research to be established, particularly around the role of obesity [10–12] but also ethnicity [13] and other factors such as comorbidities and vitamin deficiencies [14, 15].

We have developed an obesity classification system [16], with variable selection informed by the Foresight Obesity System map covering the themes of: food production, food consumption, societal influences, individual psychology, individual activity, activity environment and Biology [4], using the UKB cohort. This classification utilises 52 UKB variables composited into 23 classification variables and the k-means unsupervised classification algorithm is used to identify the classes. Pen-portraits based on how each of these classes measure against the classification variables are used to typify these seven classes as: *Active workers*; *Retirees with healthy lifestyles*; *Stressed and not in work*; *Deprived with less-healthy lifestyles*; *Comfortable professionals* and *Comfortable families*. Each class was seen to be differentiated by aspects such as gender (more males in the *Stressed and not in work* class), ethnicity (*Retirees with healthy lifestyles* were predominately white ethnicity), self-reported health (*Deprived with less-healthy lifestyles* had poorer health), and Education (*Active workers* with lower education levels).

The aim of this short communication is to investigate whether there is a relationship between our obesity classification and (a) being tested for COVID-19, (b) testing positive for COVID-19, (c) suffering acute symptoms of COVID-19 resulting in hospital admissions, or (d) suffering severe symptoms of COVID-19 resulting in death.

## Methods

We used UKB COVID-19 data up until 30 November 2020. UKB participants can be tested multiple times, and any participant with one or more positive tests is regarded as having tested positive. For hospitalisation, the ICD-10 codes of U07.1 (tested positive for COVID-19) and U07.2 (clinically diagnosed for/probable/suspected COVID-19) are used to identify hospital admissions for COVID-19. Any primary or secondary cause of death mentioning COVID-19 is recorded as a COVID-19 death. All UKB participants with our obesity classifications that were alive on the 29 January 2020, this being the date that COVID-19 cases were first detected in the UK, are included in our ‘population at risk’. World Health Organisation cut-points are used to calculate weight status categories from measured height and weight information [17].

Descriptive statistics are calculated for the relationship between our obesity classification and the four COVID-19 outcomes. As well as a consideration of how these events vary by our classification, a confirmatory analysis examines patterns in these data by obesity, gender, age, ethnicity and a measure of area deprivation.

## Results

Descriptive statistics are presented in Table 1 and the standardised differences [18] are provided in Table 2.

**Table 1:** The distribution of COVID-19 outcomes by selected UKB factors and the Foresight obesity driven classification.

From 29/01/2020 to 30/11/2020	Participants alive on 29/01/2020	% participants tested	% participants tested positive	% tests conducted that are positive	% participants hospitalised U07.X	% participants cause of death (primary and secondary) with U07.X
**All**	360,310	9.1%	1.57%	17.2%	0.36%	0.12%
**Obesity**						
Normal/under weight	123,201	7.9%	1.23%	15.4%	0.20%	0.06%
Being overweight	154,099	9.2%	1.58%	17.3%	0.35%	0.11%
Living with obesity	82,424	10.8%	2.07%	19.1%	0.60%	0.21%
Not Available	586	15.2%	1.88%	12.4%	1.37%	0.17%
**Gender**						
Male	163,138	9.6%	1.69%	17.7%	0.47%	0.17%
Female	197,172	8.8%	1.47%	16.8%	0.26%	0.07%
**Age** (on 31 January 2020)						
Aged 49 to 54	31,145	8.4%	2.67%	32.0%	0.19%	0.01%
Aged 55 to 59	49,503	8.3%	2.42%	29.3%	0.21%	0.02%
Aged 60 to 64	56,026	7.8%	1.64%	21.2%	0.19%	0.04%
Aged 65 to 69	63,927	8.1%	1.25%	15.4%	0.30%	0.07%
Aged 70 to 74	84,640	9.3%	1.06%	11.4%	0.37%	0.11%
Aged 75 to 82	75,069	11.8%	1.36%	11.6%	0.68%	0.33%
**Ethnicity**						
White	342,062	9.1%	1.54%	17.0%	0.34%	0.11%
Mixed	2133	8.4%	1.64%	19.4%	0.38%	0.05%
Asian	6256	11.4%	3.07%	26.9%	0.62%	0.14%
Black	4966	10.1%	1.79%	17.7%	0.81%	0.34%
Other	3933	8.6%	1.47%	17.2%	0.48%	0.10%
Prefer not to answer/Don’t know	960	8.4%	1.04%	12.3%	0.63%	0.21%
**Townsend**						
Least deprived quintile	75,417	8.7%	1.3%	14.6%	0.2%	0.1%
Second quintile	75,403	8.8%	1.4%	16.3%	0.3%	0.1%
Middle quintile	75,704	8.9%	1.5%	17.4%	0.3%	0.1%
Fourth quintile	75,766	9.0%	1.6%	17.9%	0.4%	0.1%
Most deprived quintile	76,850	10.3%	2.0%	19.5%	0.5%	0.2%
**Region**						
North East	41,491	11.3%	1.91%	16.9%	0.32%	0.11%
North West	55,404	10.2%	2.53%	24.7%	0.57%	0.16%
Yorkshire and the Humber	55,939	7.3%	2.13%	29.3%	0.40%	0.13%
Midlands	58,548	9.7%	1.68%	17.3%	0.42%	0.12%
South	68,648	10.6%	0.93%	8.8%	0.21%	0.07%
London	51,970	10.0%	1.00%	10.0%	0.36%	0.10%
Wales	14,739	2.1%	0.94%	44.2%	0.00%	0.11%
Scotland	13,571	0.1%	0.02%	25.0%	0.30%	0.18%
**Classification**						
Active workers	35,161	9.0%	2.24%	24.9%	0.36%	0.06%
Retirees with healthy lifestyles	77,692	9.2%	0.92%	10.0%	0.28%	0.10%
Stressed and not in work	56,679	10.2%	1.34%	13.2%	0.51%	0.19%
Deprived with less healthy lifestyles	44,624	11.8%	1.89%	16.1%	0.84%	0.38%
Comfortable professionals	66,195	8.0%	1.31%	16.4%	0.22%	0.05%
Comfortable families	79,959	7.9%	2.12%	27.0%	0.16%	0.02%

In Table 1 a larger proportion of overweight or obese people are tested, test positive and are hospitalised than those who are healthy weight. The proportions that die of/with COVID-19 is highest for those who are living with obesity (0.21% versus 0.06% for normal/underweight and 0.11% for overweight). The oldest participants (aged 70 to 82) have higher testing rates than younger participants (11.8% for the oldest group but only 8.4% for the youngest), however it is younger participants who have higher positive test rates (at 2.67%). A higher proportion of older participants are hospitalised (0.68%) and/or die with/of COVID-19 (0.33%) than younger participants (just 0.01%). Those from Asian and Black ethnic groups have a higher proportion of testing (11.4% and 10.1%, respectively) and a higher positive test rate (3.07% and 1.79%, respectively) than those from White (1.54%), Mixed (1.64%) or Other (1.47%) groups. The Black group has the highest percentage of hospitalisation (at 0.81%) and/or death (0.34%). In terms of deprivation, testing, positive results, hospitalisation and death are all higher for the most deprived quintile (10.3%, 2.0%, 0.5% and 0.2%, respectively) than the least deprived quintile (8.7%, 1.3%, 0.2% and 0.1%, respectively). In summary, for obesity, gender, age, ethnicity and deprivation, we see trends emerge where most outcomes accord with our understanding that socio-demographic [19] and socio-economic groups [20] have differing outcomes [10, 11, 21]. This provides reassurance that these data are suitable for further consideration against our obesity classification.

Our classification identifies interesting patterns. The *Active workers* class are just slightly less likely than average to get tested (at 9.0% versus 9.1%), although those that are tested are more likely than average to test positive (2.24% versus 1.57%). Whilst they have a higher percentage of ethnic minority groups, the proportion that are hospitalisation (0.36%) or die (0.06%) is low, which could be age related in this young class. Testing rates for the *Retirees with healthy lifestyle* class are just higher than average (9.2%) but the rate of positive tests are lower than average (0.92% versus 1.57%). They are also less likely than average to be hospitalised (0.28% versus 0.36%) or die (0.10% versus 0.12%). Testing rates for the *Stressed and not in work* class are high (10.2%) with hospitalisations above average (0.51% versus 0.36%), as is the proportion that die of/with COVID-19 (0.19% versus 0.12%). With the *Deprived with less-healthy lifestyles* class we see the highest testing rates (11.8%) and hospitalisation (0.84%) and death rates are the highest of any class (0.38%). Our *Comfortable professionals* class demonstrate testing rates below average (8.0% versus 9.1%), and of those tested, positive test results are also below average (1.31% versus 1.57%). This class are also less likely to be hospitalised (0.22%) or die of COVID-19 (0.05%). Testing rates for the *Comfortable families* class are the lowest in the cohort (7.9%), but of those who are tested, positive diagnoses are high (2.12%), with 27.0% of tests being positive. Here COVID-19 hospitalisation rates (0.16%) and deaths (0.02%) are the lowest of all the classes.

In Table 2 there is generally a detectable difference in the distribution of counts for the ‘treated’ and ‘not treated’ participants—demonstrating that there are differences in outcomes due to each of the four treatments. This is, however, not so much the case for gender, where there is no supporting evidence for differences both when testing, and finding a positive test outcome.

**Table 2:** Standardised differences for the categorical variables of interest (values above 0.1 are highlighted in **bold** [23]).

	% participants tested	% participants tested positive	% participants hospitalised U07.X	% participants cause of death (primary and secondary) with U07.X
Obesity	**0.130**	**0.200**	**0.415**	**0.488**
Gender	0.047	0.071	**0.301**	**0.451**
Age	**0.170**	**0.336**	**0.484**	**1.010**
Ethnicity	0.039	**0.110**	**0.156**	**0.181**
Townsend-10	0.074	**0.161**	**0.310**	**0.360**
Townsend-5	0.070	**0.156**	**0.286**	**0.348**
Region	**0.397**	**0.477**	**0.432**	**0.242**
Classification	**0.151**	**0.323**	**0.567**	**0.929**

## Discussion

Our classification, grounded in a whole systems approach to understanding obesity, differentiates COVID-19 prevalence and severity in a large UK cohort. Of significance is that neither the outcome of obesity itself or COVID-19 outcome were explicitly used in building the classification, yet this differentiating ability is present in the classification. Results show that the *Active Workers* are the most likely to test positive. This class has a high representation in manual trades who are less likely to be able to work from home during the pandemic and have busy lives, making them less able to adapt. They are, however, healthy enough as a group that a lower proportion are hospitalised with or die from/with COVID-19 (this group have a high percentage who self-report no doctor diagnosed illnesses). The *Retirees with healthy lifestyle* are a class that looks after themselves well and generally have access to the resources which would facilitate the ability to isolate. They have the knowledge and time to be able to get tested, but also demonstrate low positive rates. Being an older demographic who were advised by the government to ‘shield’, avoids contacts and potential infections, and so they are less likely to test positive, suffer hospitalisation or die. A different picture emerges for *Stressed and not in work*, where it is possible that these participants present with symptoms or are being tested because they are undergoing other medical treatments (this is one of the two classes that report poor general health and the presence of long standing illnesses—including diabetes and cancer) and would therefore be picked-up through routine testing at hospital admission. Those in this class who do have COVID-19 are more likely to be hospitalised or die.

Another class with poor outcomes is *Deprived with less-healthy lifestyles*. These participants may generally lack the necessary societal and economical resources to self-isolate effectively. Additionally, a reason for the high testing rates here could be due to presenting with symptoms or screening as part of other treatments (as was the case for the *Stressed and not in work*). Their standard of living and older age profile account for high hospitalisation and mortality. The *Comfortable professionals* class is largely composed of younger, busy individuals and these participants are less likely to get tested. A proportion of these participants have higher qualifications and are employed in professional occupations and many will be working from home, with less exposure. They also live in smaller households, so have a lower likelihood of within household transmission, and in less deprived areas, where rates are lower. *Comfortable families* are a relatively healthy group who also live in less deprived areas. Given the characteristics of this group any symptoms might not be severe and their testing rates are the lowest of all classes. They did however live in larger households, likely at one time to be containing children or young adults, so the within home transmission may explain the higher positive rates for those who are tested. Lifestyle, age and health may explain hospitalisation and death being low.

As we learn more about risk factors for COVID-19 we begin to understand that they incorporate a complex interplay of a range of biological, social and environmental risks, in a similar manner to obesity. This communication presents a classification tool that is able to highlight at risk groups in the same way that it can distinguish those most likely to be overweight or living with obesity. This may be through obesity acting as mediator for COVID-19 or that the classification captures latent variables that are underlying risk factors for both obesity and COVID-19.

There are known biases in the UKB data which limit the wider generalisability of findings, given that the population is generally white, middle aged and more affluent [22]. That said this work presents an important proof of principle that could be replicated elsewhere. With future data releases, follow up work could investigate whether the classification can be used in understanding long-COVID risk.
